# Research on the Fastest Detection Method for Weak Trends under Noise Interference

**DOI:** 10.3390/e23081093

**Published:** 2021-08-22

**Authors:** Guang Li, Jing Liang, Caitong Yue

**Affiliations:** 1 School of Electrical Engineering and Automation, Henan Institute of Technology, Xinxiang 453003, China; lg@hait.edu.cn; 2 School of Electrical Engineering, Zhengzhou University, Zhengzhou 450001, China; zzuyuecaitong@163.com

**Keywords:** data stream, sliding window, machine learning, window length

## Abstract

Trend anomaly detection is the practice of comparing and analyzing current and historical data trends to detect real-time abnormalities in online industrial data-streams. It has the advantages of tracking a concept drift automatically and predicting trend changes in the shortest time, making it important both for algorithmic research and industry. However, industrial data streams contain considerable noise that interferes with detecting weak anomalies. In this paper, the fastest detection algorithm “sliding nesting” is adopted. It is based on calculating the data weight in each window by applying variable weights, while maintaining the method of trend-effective integration accumulation. The new algorithm changes the traditional calculation method of the trend anomaly detection score, which calculates the score in a short window. This algorithm, SNWFD–DS, can detect weak trend abnormalities in the presence of noise interference. Compared with other methods, it has significant advantages. An on-site oil drilling data test shows that this method can significantly reduce delays compared with other methods and can improve the detection accuracy of weak trend anomalies under noise interference.

## 1. Introduction

Data stream trend anomaly detection, a branch of data-flow research, has been used in online real-time data in the following fields: intelligent engineering [1] and systems using automated detection [2], oil drilling [3], smart meters [4], e-commerce [5], and household energy consumption monitoring [6]. The above data flow trend has the following characteristics: one scan, concept drift, data time series, unpredictability, high-speed data updates, and unlimited data. However, industrial data is often accompanied by noise caused by the working environment or the sensor, which impairs trend anomaly detection and can lead to misjudgments.

Many scholars have carried out studies on the difficulty of detecting trend anomalies under noise interference. For example, Li [3] adopted the sliding nested window technique, which weakened the weight of data, reduced the influence of noise, and significantly improved the identification of abnormal trends in a noisy environment; Cavaglia [7] used a random forest and genetic algorithm to analyze noisy gravitational waves. This method successfully identified non-astrophysical data and helped eliminate noise. Hasan [8] used reverse indexing and incremental clustering to mine noisy Twitter data streams using the smallest amount of calculation data and classified them into major and minor events. The recall rate and accuracy of this method are both significant improvements. Heitor [9] proposed classifying data streams using an adaptive random forest (ARF) algorithm, which improved on traditional data stream trend anomaly detection methods.

The online data stream is incremental and based on endless data, so it is necessary to intercept the current moment and a small piece of historical data to be the object of analysis. Many real-time processing models have often used a sliding window length of 1, in which only the data of all parameters at the current moment is used, not historical data. For example, Tang [10] used deep CNNS to control wireless network traffic. The application of deep learning to control the data at the current moment slowed the packet loss rate. This method also has no application to historical data and only controls multiple parameters at the moment. Athanasios [11] used a new real-time, deep-learning method to train a model using multiple sensors to improve a robot’s generalizability, adaptability, and convergence. The data used was still the current time data of multiple parameters; historical data was not used. Mao [12] used deep-learning to control the adaptive resource ability of the software-defined networking communication system. Through online training, the amount of calculation by the central processing unit was reduced. Meyer [13] also used deep-learning methods (circulating neural networks) to predict serious real-time complications after cardiac surgery (mortality, renal failure, postoperative bleeding), using multiple sensors to detect the organ data in real time. The machine-learning indicator area under the curve (AUC) was significantly better than the clinical indicators; however, this method used multiple parameters but not all of them applied in this case. The historical data of the parameters were used effectively. Xu [14] used offline data to learn classification rules and trained random forest models using machine-learning methods. This method presented the real-time realization of a dynamic programming algorithm for power optimization in an organic Rankine cycle waste heat recovery system. It used multiple parameters but not historical data.

Noise, though, makes the detection of trend anomalies difficult. For example, Thomson [15] has four 100 Hz power spectral density sensors installed at the bottom of the ocean, as well as an automatic identification system (AIS), and shipping and trade statistics to describe the effect of a new coronary pneumonia on global trade. The article described a trend analysis, but it was based on noisy sensor data. The research is still the current data, and no historical data was intercepted and analyzed.

The above literature shows that the detection of data-flow trend anomaly in noisy environments is widely used in industry, aerospace, and other fields. Based on the sliding nesting algorithm, this paper adopted the forgetting strategy to build a trend anomaly detection algorithm under the influence of incremental data-stream noise and improved the timeliness of algorithm detection without affecting the accuracy of trend anomaly detection. The flow trend was abnormal to ensure the accuracy and timeliness of the real-time online data-flow analysis.

This article first introduces the research background and the problems to be solved. Then it proceeds to discuss the trend anomaly detection algorithm and the problems to be solved, followed by the verification of the algorithm using a real industrial data stream and conclusions.

## 2. Related Work

### 2.1. Sliding Window

The sliding window is commonly used to process data streams. It is a window of a pre-established length that moves as new (current) data enters from one end and pushes old (historical) data out the other. In this way, current and historical data are always changing, and this affects the data being analyzed. For example, Wang [16] proposed a Gaussian restricted Boltzmann machine (GRBM) model that supported decimal input using a sliding window and trained it through iterations. At the same time, he proposed a method based on the Kullbeck–Leibler divergence (KL distance). A data-flow adaptive block algorithm compared the probability distribution differences of sliding windows. Finally, the predicted value was obtained according to the distribution of the previous data and determined if the KL distance was within the confidence interval to realize the adaptive adjustment of the sliding window and the division of the data stream.

In addition to the sliding window, there are two other commonly used methods to processing data streams. One is the landmark model. It uses the memory of important moments and uses each moment as a calibration point to analyze the data that comes after it, as proposed by Leung [17]. A landmark model mines frequent patterns from uncertain data streams. Because it needs to mark important moments from which to start calculating future data, it also needs to identify important marks when the subsequent data ends. Therefore, the mark data becomes a very important factor in determining the effect of this method, which means that for data streams without important marks, this method is of limited value.

The other method is the snapshot model, which intercepts and analyzes the data stream. For example, Krämer [18] developed a general method for dynamic migration planning by treating the analyzed data as a black box, equivalent to a snapshot. This enabled the query optimizer to apply regular conversion rules during optimization. This method supports the dynamic optimization of arbitrary continuous queries that can be expressed in CQL.

**Figure 1:**
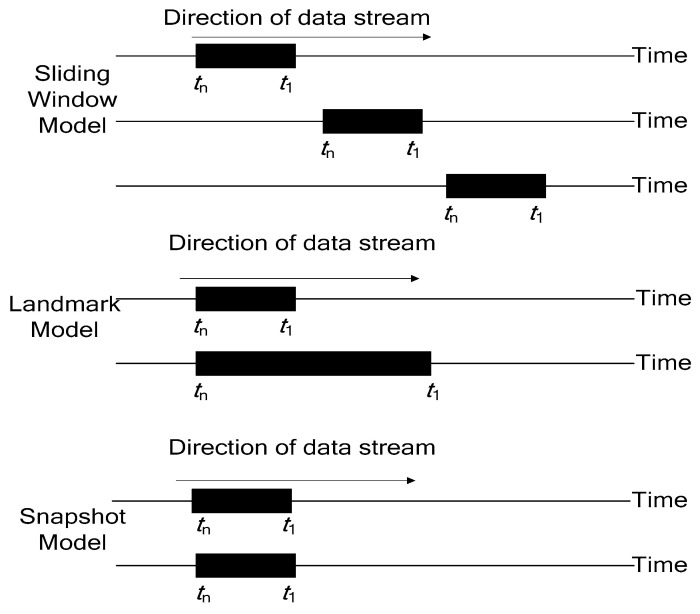
Data stream processing based on different windows.

Figure 1 shows the characteristics of the three window models. For the sliding window, the processing data range remained unchanged. It always used current-time data and similar fixed-length data. According to the above analysis, the weights of the historical and current data remained unchanged as the dynamic data stream was turned into a static data analysis. The data in the window closest to the current moment was often the most valuable for research and is usually the focus of user attention. The landmark model also turned a dynamic stream static, and as the amount of data processed increased, the algorithm needed to respond promptly to new data. With the continuous arrival of new data, the amount of analyzed data increased. Regarding the snapshot model, data analysis was fixed to the length of the data stream and immediately followed the current time data.

The sliding window is the most commonly used data stream processing model because it meets the requirements of real-time data analysis and consider both current data and historical data. It is even used in the noise environment of oil drilling because it can detect abnormal changes in trends in the presence of noise interference. Because valuable information is hidden under the noise, real-time data filtering has become a prerequisite. The sliding window nesting method divides the historical period into short-time and relatively long-time windows. Two types of secondary variable parameters, or multiple derivative variable parameters, are calculated in each, and target detection is calibrated through these extracted feature parameters. For example, Du [19] accurately detected small targets against a complex background and under low signal-to-noise ratio conditions. Because it is difficult to detect small and weak targets against a complex background using local and overall contrast methods, a small-target detection method based on facet-kernel filtering has been shown to enhance small targets while suppressing complex background clutter.

The facet-kernel filter enhances the center layer target, while the background is suppressed by calculating the grey similarity difference between the center and surrounding layers. Li [20] proposed a residual fusion network (RFN) to be trained by a new loss function with preserved details and a loss function with enhanced features. In the first of this new two-stage training strategy, an autoencoder is trained based on an innovative nested sliding window. Next, the RFN is trained using the proposed loss function. Experimental results on public domain datasets show that, compared with existing methods, this method is subjectively and objectively superior to existing methods.

**Figure 2:**
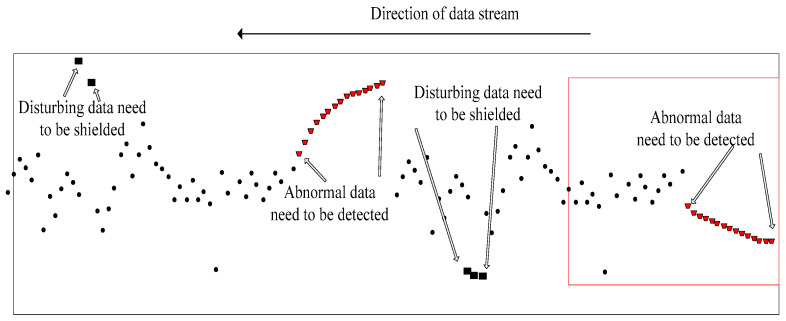
Sliding nest window trend anomaly detection schematic diagram.

The so-called sliding nested window is a small sliding window nested inside a larger one. As shown in Figure 2, the data points of the large and small windows overlap at the current moment, and the historical data range is different. Sliding nested windows can bring benefits by weakening the impact of the current point and increasing trend analysis. Because the small window is not long, it can reflect real-time characteristics of data flow changes and block the interference of the few data that deviate from the trend to preserve the overall trend. The front-end and middle data that deviated from the normal trend quickly returned to the overall data change. Therefore, these data are interference data and should not be classified as abnormal; otherwise, it will lead to wrong decisions. However, the back-end and middle segments of the data continued to deviate from the overall trend. These were regarded as abnormal data, so abnormal point and degree detection were required. These data were labelled according to the changes, and from this classification, several decisions were made based on an abnormal parameter. The abnormal data that needed to be detected rose or fell for internal reasons, and this should be our target because the abnormal changes of these data are consistent with our decision-making classification goal. Therefore, reducing misclassification caused by data randomness increases the trend judgment and improves the forecast accuracy.

According to its algorithm, trend anomaly detection can be known only after coming in contact with several data with abnormal trends. Therefore, for real-time systems, the shorter the time it takes to determine an abnormal trend the better. However, due to the limitations of the trend anomaly detection algorithm, especially for interference data, the accuracy of detection cannot be guaranteed because of the gradual-trend anomaly interference noise. In order to ensure detection accuracy, the sliding window is combined with other trend anomaly detection algorithms.

More and more applications, such as Riss [21] proposed an automatic near-real-time fault identification method based on a combination of sliding window and cumulative sum CUSUM control charts; that is, the combination of CUSUM charts and identification system (HC–ERS); this method has proved to be effective, achieving a high true detection rate of 82% and a low false alarm rate of an average of 0.3 per week. This method showed the ability to detect a WTW failure. Therefore, it has the potential for practical application in the water industry. Li [22] used the combination of two CUSUM charts and sliding windows to provide support for the detection of weak-trend oil-drilling data flow under noise interference.

**Figure 3:**
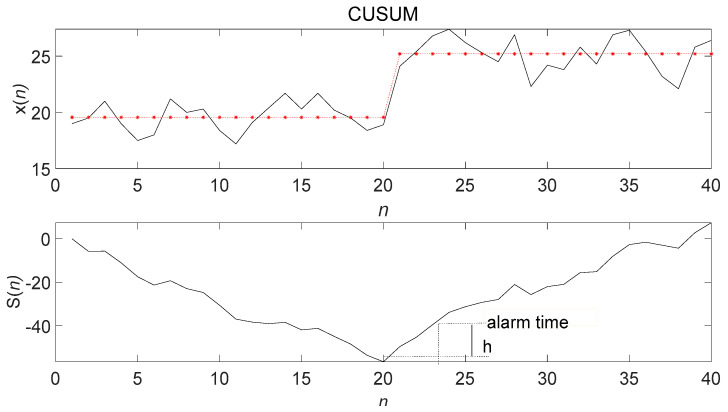
Schematic diagram of CUSUM control chart.

The above trend abnormality detection methods all faced the same problem: timeliness of detection; that is, the distance between the time that the trend is detected and the current moment. Figure 2 shows that the data on the far right was the current-time data. As time went by, new data continuously entered the sliding window from the far right. Old data continuously left from the far left, thereby realizing data flow. The limitations of the algorithm and a certain historical data trend change determined the abnormal trend, as shown in Figure 3. The changed data point had a certain delay from the current moment. Therefore, overcoming the trend anomaly delay problem, similar to CUSUM, by shortening the determination delay as much as possible and meeting the timeliness of the data flow are the challenges of research.

### 2.2. Extrapolation of Online Data Segmentation

The push-back continuation is a common method for trend anomaly detection. It uses the current time to delay the period of calculating the current trend change. Meng [23] used the residential electricity consumption and population data of 30 provinces from 2001 to 2014 for a three-dimensional decomposition model and a hybrid trend extrapolation model to explore the driving factors of China’s growing residential electricity consumption and predict what it will be by 2030. Colonna [24] used the continuation method to estimate the prevalence rate in France in 2002 and 2012 and the prevalence trend during 1993–2002. Based on analyzing the existing radar extrapolation trend model, Gan [25] improved it by increasing the number of radar layers and the weight of the prediction results. Experiments were conducted with public competition data and real radar data as samples. The results showed that the improved network model captured spatio-temporal correlation better and had more accurate extrapolation. Ma [26] proposed a new spatial interpolation/extrapolation method to generate the spatial distribution of atmospheric pollutant concentrations. The model is developed based on a long short-term memory (LSTM) neural network to capture the long-term dependence of air quality, and a geological layer was designed to integrate the temporal and spatial correlations of other monitoring stations. To evaluate the effectiveness of the proposed method, a case study was conducted in Washington State. The experimental results show that the root mean square error (RMSE) of the new method was 0.0437, nearly 60.13% higher than that for the traditional method.

**Figure 4:**
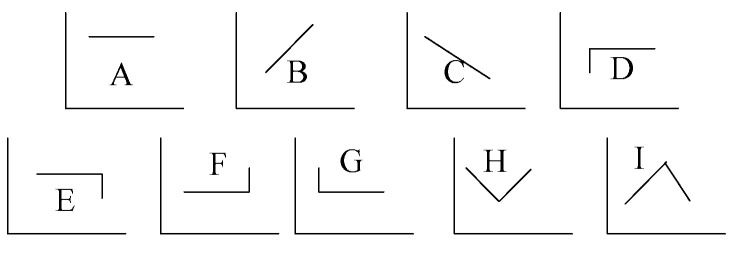
Primitives trend set.

It can be seen from Figure 4 that trend anomaly detection can be divided into nine categories. In engineering, trend types E and F are often used because there is not much interesting information in normal trend changes. Abnormal changes often correspond to engineering accidents because capturing, so this information is of great significance for engineers.

**Figure 5:**
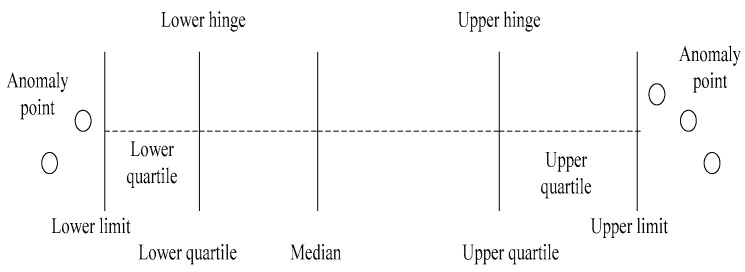
Parameters of Box Plot.

As shown in Figure 5, the box plot, a method of online data flow labelling, analyzes data that exceeds the upper and lower limit curves and continuously monitors the upper and lower abnormal boundary values online to make determinations about the data. The advantage of this method is that it tracks the dynamic trend of the data stream and classifies the data in the sliding window in real-time. Based on meeting the concept drift, it can adapt in real-time to changes of parameters, and the sliding window can meet the requirements of the data stream.

From the above analysis, the trend anomaly detection algorithm does not consider the time of the trend anomaly because detection has a paradox within a certain range; that is, the shorter the detection time, the lower the detection accuracy, but the reverse is not true. The restricted is the result of the design of the algorithm and the processing data environment. Therefore, discussing the accuracy of the algorithm and the detection time is an important aspect of trend anomaly detection.

## 3. Sliding Nested Fastest Trend Anomaly Detection Algorithm

It can be seen from Figure 2 that the purpose of trend anomaly detection is to detect weak trend anomalies under noise interference, which often leads to misjudgments. Therefore, the nested sliding windows method removes noise interference to calculate the current segment mean value and variance over time and the changes in time and variance over a long period. However, because of the design of the algorithm the anomaly detection time has a large lag.

The new algorithm still uses the sliding nesting method, but the data weights in the two windows are updated and changed in real-time: the closer the data to the current time the greater the weight; the farther the data, the smaller the weight.

(1) $$y(t)=p(t-t_{0})+y_{0}$$

where ($t_{0}$, $y_{0}$) is the interval of the starting point, and *p* is a slope.

In the original and prediction models, (Formulas (2) and (3), respectively) the initial time is $t_{b0}$, the initial result value is $y_{b0}$, the slope is $k_{0}$, and Δt is the update interval.

(2) $$y(t)=k_{0}(t-t_{b0})+y_{b0}$$

(3) $$\overset{\wedge }{y}(t_{0}+m\Delta t)=k_{0}(t_{0}+m\Delta t-t_{so})+y_{so}$$

The deviation between the two is the benchmark for trend anomaly detection, as shown in Formula (4).

(4) $$e(t_{0}+m\Delta t)=y(t_{0}+m\Delta t)-\overset{\wedge }{y}(t_{0}+m\Delta t)$$

The sum of the cumulative deviations in the two windows of length is shown in Formula (5).

(5) $$cusum(t_{0}+m\Delta t)=cusum(t_{0}+(m-1)\Delta t)+e(t_{0}+m\Delta t)=\sum_{j=0}^{m}e(t_{0}+j\Delta t)$$

In Formulas (6) and (7), the stop time is $t_{b}(i)$; the corresponding output is $y_{s}(i)$; and T is the time interval. The formulas are

(6) $$t_{b}(i)=t_{s}(i+1)-T$$

(7) $$y_{s}(i)=p(i)[t_{s}(i)-t_{b}(i)]+y_{b}(i)$$

The determination formula for weak trend abnormality is

(8) $$I(i)=y_{s}(i+1)-y_{s}(i)=\{p(i+1)[t_{s}(i+1)-t_{b}(i+1)]+y_{b}(i+1)\}-\{p(i)[t_{s}(i)-t_{b}(i)]+y_{b}(i)\}$$

The formula for abnormally drastic changes in the trend is

(9) $$I_{D}(i)=y_{b}(i+1)-y_{s}(i)=y_{b}(i+1)-\{p(i)[t_{s}(i)-t_{b}(i)]+y_{b}(i)\}$$

The formula for abnormal changes in the later trend is

(10) $$I_{b}(i)=y_{s}(i+1)-y_{b}(i+1)=p(i)[t_{s}(i+1)-t_{b}(i+1)]$$

**Figure 6:**
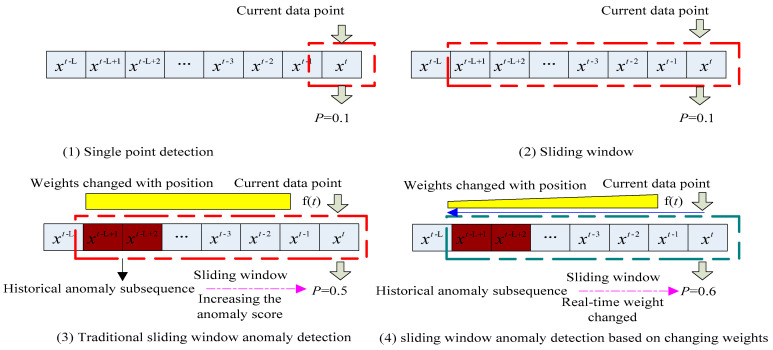
Sliding window-based anomaly detection with changed weights.

From the basic formula of the above trend, the sliding nested window fastest detection of data stream (SNWFD–DS) algorithm was based on the sliding nest window chart anomaly detection, which was based on the data stream algorithm (SNWCAD–DS) [3], which is an improvement on the double CUSUM-based data stream (DCUSUM–DS) [22] algorithm. Instead of the cumulative sum, the sliding nested window algorithm was adopted, and variable weight was the strategy [26] to update the data as shown in Figure 6.

Algorithm 1 is the algorithm of real-time weight transformation.
**Algorithm 1** Sliding Nest Window Weights Changed Algorithm**SNWWCA:** initialize LW, SW
Set weights range: (*a*, *b*)
Compute weights step: (*b* − *a*)/LW; (*b* − *a*)/SW;
Update: *t*;
Update: P * (*b* − *a*)/LW; S * (*b* − *a*)/SW;
Update: P * (*b* − *a*)/LW; S * (*b* − *a*)/SW;

LW is the length of the long sliding window; SW is the length of the short sliding window; T is the number of anomalies; β is the number of anomaly determinations; $medL_{x}$ is the long window mean; $medS_{x}$ is the short window mean; $stdL_{x}$ is the long window variance; $stdS_{x}$ is the short window variance; $Dif_{-}ma_{-}meanS_{Dif_{-}ma}$ is the mean of the difference between the long and short means in the short window; $Dif_{-}ma_{-}meanL_{Dif_{-}ma}$ is the mean of the difference between the long and short means in the long window; $Dif_{-}ma_{-}stdS_{Dif_{-}ma}$ is the variance of the difference between the short and long mean in the short window; and $Dif_{-}ma_{-}stdL_{Dif_{-}ma}$ is the variance of the difference between the short and long mean in the long window.

It can be seen that Algorithm 2, SNWFD–DS, performed two average calculations: $Dif_{-}ma_{-}meanS$ is the mean in the short window of $Dif_{-}ma_{-}result$, and $Dif_{-}ma_{-}meanL$ is the mean in the long window of $Dif_{-}ma_{-}result$. $Dif_{-}ma_{-}stdS$ is the variance within the short window of $Dif_{-}ma_{-}result$; and $Dif_{-}ma_{-}stdL$ is the variance within the long window of $Dif_{-}ma_{-}result$. SNWFD-DS used two calculations of the mean and variance. Based on the two mean variances, only the deviation from the calculated amount at the current moment was calculated. Then, the score of the current moment data was given, and the score in a short period was calculated. If the score was high, it represented the current moment.
**Algorithm 2: SNWFD-DS****DCUSUM-DS:** initialize LW, SW, T, β
Compute weights of SW and LW
Compute: $medS_{x}$, $stdS_{x}$, $medL_{x}$, $stdL_{x}$
$Dif_{-}ma=medS_{x}-medL_{x}$
Compute: $Dif_{-}ma_{-}meanS_{Dif_{-}ma}$, $Dif_{-}ma_{-}meanL_{Dif_{-}ma}$, $Dif_{-}ma_{-}stdS_{Dif_{-}ma}$, $Dif_{-}ma_{-}stdL_{Dif_{-}ma}$
$Dif_{-}ma_{-}result=Dif_{-}ma_{-}meanS_{Dif_{-}ma}-Dif_{-}ma_{-}meanL_{Dif_{-}ma}$
Compute: $Dif_{-}ma_{-}meanS$, $Dif_{-}ma_{-}meanL$, $Dif_{-}ma_{-}stdS$
Whether $Dif_{-}ma_{-}result>0$
Compute: $\mathrm{sum}(Dif_{-}ma_{-}result)$
Compute: Result=Dif−ma−meanL*abs(sum(Dif−ma−result))
Box(Score(Result))
Whether Score > β
Output: label VA

## 4. Real Data Verification

The data used in this paper came from the Tarim Oil field. The specific parameters were the total pool volume relative to the gradual change in the trend of the drilling leakage overflow. The total pool volume sensor detected the height of the total pool volume and was affected by surroundings. There was noise and other influences in the working environment, so detecting the gradual trend of parameters such as the total pool volume was the objective. The program software used in this article was matlab2020a, the CPU was Intel(^®^) Core(™) i5-4570 @ 3.2 GHz 3.2 GHz, the memory was 16 G, and the operating system was Window10 64-bit.

As shown in Table 1, to improve the fairness and credibility of each algorithm’s detection accuracy, the same parameters for the false alarm rate and algorithm complexity were set for each algorithm. The long window was uniformly set to 400, the short window set to 80, the threshold set to 0.4, and the outbound rate set to 8. The weight size was the same for the first two algorithms, but the new algorithm used a variable weight setting, that was constantly updated with time. The following results, in addition to the data listed in Table 2, used the parameter settings in Table 1 and compared the advantages and disadvantages of each algorithm by comparing the TPR, FPR, the AUC, and the algorithm calculation time.

**Figure 7:**
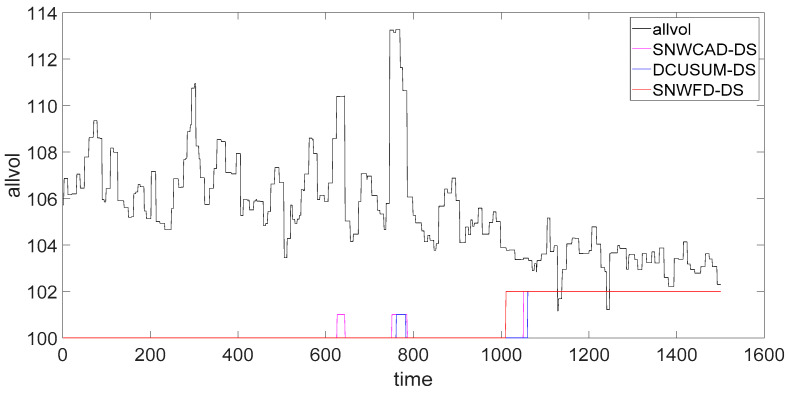
Comparison of classification results.

As shown in Figure 7, the abnormal rising flag is 101, and the abnormal falling flag is 102. Through the real data comparison of the three methods, we saw that the SNWFD–DS not only detected the weak abnormal trend decrease but also shielded the abnormal rise caused by noise interference. From the time of abnormal warning, SNWFD–DS detected the trend better than the SNWCAD–DS or DCUSUM–DS did. The abnormal time is early and was advanced by about 40 s.

**Figure 8:**
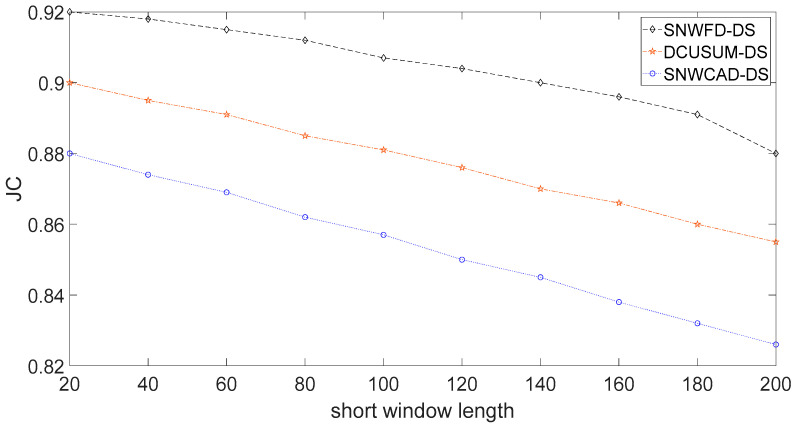
Jaccard’s coefficient of each algorithm.

Figure 8 shows the distribution of Jaccard’s coefficient. The abscissa is the length of the short window. The long window length was fixed at 400. This length means that the impact of different short window lengths on each algorithm was compared in about 7 min. The meaning of this expression is to obtain different Jaccard’s coefficients by setting different window lengths and to compare the quality of the algorithm. Jaccard’s coefficient represents a similarity measure of the online stream classification algorithm for data stream machine learning. The factors affecting the coefficient include not only the classification result, error, and missing report rates but also the setting of the window length and the outbound rate. Due to the delay, the higher Jaccard’s coefficient, the higher the accuracy.

**Figure 9:**
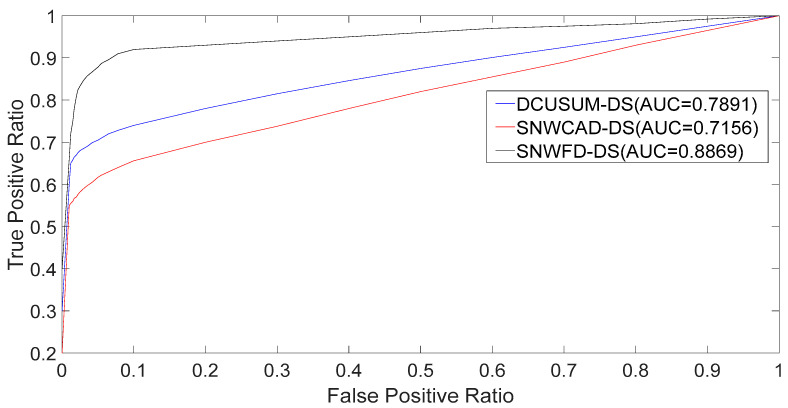
The receiver operating characteristic (ROC) curve and the area under the curve (AUC).

Table 2 is a further interpretation of Figure 9. It shows the time of each single-step analysis for different lengths of the short window and objectively evaluates the complexity of the algorithm by averaging the time. According to the sampling frequency of 1 Hz, the proposed new algorithm was calculated within 0.2 s in a 1 s interval, which fully met the operating requirements of the site. Compared to the DCUSUM–DS algorithm, the SNWFD–DS had an additional variable weight calculation, so the calculation increased. In addition, when calculating the final score, the number for the SNWFD–DS was slightly higher due to different selection strategies.

It can be seen from Figure 9 that, compared to the SNWCAD–DS and DCUSUM–DS, the SNWFD–DS algorithm had a higher AUC because of the different weighting and final score selection strategies.

The data in Table 3 show the length of the long window fixed of 400, and the length of the short sliding window as changed. The trend anomaly discrimination time of the three algorithms was compared with the real trend anomaly time, and the length of the delay was compared. The comparison showed that the SNWFD–DS algorithm had a shorter time delay than did the SNWCAD–DS or DCUSUM–DS algorithms. Even though the trend anomaly algorithm had a delay, the average of 10 s fully met the complexity of oil drilling engineering accidents with slow changes in trends. When there are signs of complex engineering accidents (10 s after an abnormal trend occurs) it can be predicted without their further deterioration.

## 5. Discussion

From the analysis of the figures and tables in the fourth section, the new algorithm SBWFD–DS had higher anomaly detection accuracy and shorter time delay compared to DCUSUM–DS. When the SNWFD–DS algorithm was not lower than the DCUSUM–DS in trend anomaly detection performance, it shortened the time for determining the trend anomaly. Although SNWFD–DS was compared with SNWCAD–DS, the delay time did not have obvious advantages, but the accuracy of trend anomaly detection was significantly higher than that of SNWCAD–DS. From the above analysis, the newly proposed algorithm SNWFD–DS had obvious trend anomaly detection accuracy and had the shortest time delay. It not only met the requirements of on-site trend anomaly detection, but also met the characteristics of on-site real-time operation.

The algorithm proposed in this article was verified with real data from dozens of wells in the Tarim Oil field, and the results are shown in Section 4, which shows the effectiveness of the method.

The trend anomaly detection algorithms listed in this article had to meet certain constraints. The degree of anomaly trend could not be too weak because a weak trend requires a long time, and the amount of data to be calculated for each engineering parameter was relatively large. However, there were hundreds of engineering parameters on site, and the calculation of so many parameters prevented the on-site computer from working. Limited by the calculation conditions, the long window of a single parameter of oil drilling is limited to 500 pieces of data. Therefore, very weak trend anomalies could not be detected by the algorithm proposed in this article. In addition, the detection time of the trend anomaly cannot be overly short because often that leads to the inability to distinguish between noise interference and a true trend anomaly.

Because the trend anomaly detection of the oil drilling data stream was the thinking mode of the simulated field experts, the algorithm proposed in this article was applicable to the engineering, mud, and gas measurement parameters for oil drilling. However, as the position of the sensor moved downward, more and more sensors were installed near the drill bit, and more and more sensors with higher sampling frequencies appeared. Whether the algorithm in this article was effective at detecting trend anomalies remains to be seen.

## 6. Discussion, Implication, and Conclusions

The fastest detection algorithm of sliding nesting was adopted. Based on the principle of sliding nesting, the data in each window was calculated with variable weights––higher for the data at the current moment and then reduced with increasing distance––which greatly reduced the complexity of the algorithm, and shortened the time for detecting weak trend abnormalities under noise interference by about half. This strategy can sensitively detect trend changes at the current moment, and can effectively eliminate noise interference. This method can improve the accuracy of trend anomaly detection. Compared with the previous trend anomaly detection method, this method adopts the integration method, which can make the trend anomaly detection be detected faster. Adopting a weighted data strategy improved prediction accuracy; the point of accumulation and function integral to data accumulation was retained; the method of abnormal judgment was changed; the forecasting time was advanced; and the timeliness of forecasting was ensured.

The new algorithm proposed in this paper can improve the accuracy of trend anomaly detection under noisy environments, and reduce the detection time. Faint abnormal trends often correspond to various engineering accidents or changes in working conditions. If these engineering accidents are not dealt with in time, they will often have very serious consequences or produce more difficult problems to deal with. For example, the occurrence of overflow in oil drilling is manifested as a slight decline in the outlet flow. If the weak anomaly of the outlet flow trend is not detected and no further treatment is performed, it will cause a blowout, or even cause damage to the derrick, or death. Thus, the fastest detection time can give more time for later processing. Therefore, the algorithm in this paper is of great significance to reduce equipment damage and deaths in industrial production.

The trend anomaly algorithm mentioned in this article has some limitations. It is limited by the characteristics of the real-time trend anomaly detection algorithm, which is a general problem of trend anomaly detection to detect the abnormal change of the trend in the shortest time period possible. Therefore, the amount of data processed in a single time is within the ability of the computer, but the algorithm must be able to reflect the change of the trend. Therefore, the algorithm proposed in this article is powerless for particularly slow abnormal changes in the trend. Second, particularly sensitive trend changes are often drowned in noise. Therefore, the trend anomaly detection algorithm proposed in this article is invalid for particularly sensitive trend anomaly detection.

Features correspond to various engineering accidents or status changes, so feature extraction in industrial data streams is very important. Because the data stream has the characteristics of real-time update and concept drift, the feature extraction of traditional static data will no longer be used. This article is aimed at the trend anomaly detection of a single data stream; however, in industrial production, engineering accidents or status changes often correspond to the trend anomaly detection of multiple data streams. Our next work will concern the selection method for machine-learning features based on concept drift, research on the reduction methods for high-dimensional data streams, and the applicability of original data features to deep-learning algorithms.

## Figures and Tables

**Table 1:** Algorithm setting table.

Algorithm	Length of Short Window	Length of Long Window	Threshold	Out Rate	Weights
SNWCAD–DS	80	400	0.4	8	0.0125/0.0025
DCUSUM–DS	80	400	0.4	8	0.0125/0.0025
SNWFD–DS	80	400	0.4	8	(0:0.003:0.0253)/ (0:0.0001:0.005)

**Table 2:** Computational complexity comparison.

Setting of Short Window	DCUSUM-DS	SNWCAD-DS	SNWFD-DS
20	0.2718	0.2254	0.2931
40	0.2823	0.2391	0.3072
60	0.2919	0.2508	0.3246
80	0.3077	0.2640	0.3384
100	0.3204	0.2764	0.3565
120	0.3358	0.2912	0.3718
140	0.3441	0.3047	0.3867
160	0.3586	0.3269	0.3996
180	0.3760	0.3416	0.4129
200	0.3862	0.3587	0.4276
average	0.3238	0.2879	0.3618

**Table 3:** Algorithm calculation result delay time.

Setting of Short Window	SNWCAD-DS	DCUSUM-DS	SNWFD-DS
20	9	11	8
40	12	22	10
60	14	31	12
80	15	42	13
100	18	53	15
120	17	62	16
140	20	74	17
160	23	82	19
180	25	93	20
200	28	105	21
average	18.1	57.5	15.1

## Data Availability

Not applicable.

## References

[1] Wu W., He L., Lin W. (2019). Local trend inconsistency: A prediction-driven approach to unsupervised anomaly detection in multi-seasonal time series. arXiv.

[2] Chen R.Q., Shi G.H., Zhao W.L., Liang C.H. (2019). A Joint Model for Anomaly Detection and Trend Prediction on IT Operation Series. arXiv.

[3] Li G., Wang J., Liang J., Yue C. (2018). Application of sliding nest window control chart in data stream anomaly detection. Symmetry.

[4] Yassine H., Ghanem K., Alsalemi A., Bensaali F., Amira A. (2021). Artificial intelligence based anomaly detection of energy consumption in buildings: A review, current trends and new perspectives. Appl. Energy.

[5] Gao J., Song X., Wen Q., Wang P., Sun L., Xu H. (2020). RobustTAD: Robust time series anomaly detection via decomposition and convolutional neural networks. arXiv.

[6] Liu X., Lai Z., Wang X., Huang L., Nielsen P.S. (2020). A Contextual Anomaly Detection Framework for Energy Smart Meter Data Stream. Proceedings of the International Conference on Neural Information Processing.

[7] Cavaglia M., Staats K., Gill T. (2018). Finding the origin of noise transients in LIGO data with machine learning. arXiv.

[8] Hasan M., Orgun M.A., Schwitter R. (2019). Real-time event detection from the Twitter data stream using the TwitterNews+ Framework. Inf. Process. Manag..

[9] Gomes H.M., Bifet A., Read J., Barddal J.P., Enembreck F., Pfharinger B., Holmes G., Abdessalem T. (2017). Adaptive random forests for evolving data stream classification. Mach. Learn..

[10] Tang F., Mao B., Fadlullah Z.M., Kato N., Akashi O., Inoue T., Mizutani K. (2017). On removing routing protocol from future wireless networks: A real-time deep learning approach for intelligent traffic control. IEEE Wirel. Commun..

[11] Ribeiro D., Mateus A., Miraldo P., Nascimento J.C. A real-time deep learning pedestrian detector for robot navigation. Proceedings of the 2017 IEEE International Conference on Autonomous Robot Systems and Competitions (ICARSC).

[12] Mao B., Tang F., Fadlullah Z.M., Kato N. (2019). An intelligent route computation approach based on real-time deep learning strategy for software defined communication systems. IEEE Trans. Emerg. Top. Comput..

[13] Meyer A., Zverinski D., Pfahringer B., Kempfert J., Kuehne T., Sündermann S.H., Stamm C., Hofmann T., Falk V., Eickhoff C. (2018). Machine learning for real-time prediction of complications in critical care: A retrospective study. Lancet Respir. Med..

[14] Xu B., Rathod D., Yebi A., Filipi Z. (2020). Real-time realization of Dynamic Programming using machine learning methods for IC engine waste heat recovery system power optimization. Appl. Energy.

[15] Thomson D.J.M., Barclay D.R. (2020). Real-time observations of the impact of COVID-19 on underwater noise. J. Acoust. Soc. Am..

[16] Wang W., Zhang M. (2020). Data Stream Adaptive Partitioning of Sliding Window Based on Gaussian Restricted Boltzmann Machine. Artificial Intelligence in China.

[17] Leung C.K.S., Jiang F., Hayduk Y. A landmark-model based system for mining frequent patterns from uncertain data streams. Proceedings of the 15th Symposium on International Database Engineering & Applications.

[18] Krämer J., Yang Y., Cammert M., Seeger B. (2006). Dynamic plan migration for snapshot-equivalent continuous queries in data stream systems. Proceedings of the International Conference on Extending Database Technology.

[19] Du P., Hamdulla A. (2019). Infrared Small Target Detection Based on Facet-Kernel Filtering Local Contrast Measure. Proceedings of the China Conference on Wireless Sensor Networks.

[20] Li H., Wu X.J., Kittler J. (2021). RFN-Nest: An end-to-end residual fusion network for infrared and visible images. Inf. Fusion.

[21] Riss G., Romano M., Memon F.A., Kapelan Z. (2021). Detection of water quality failure events at treatment works using a hybrid two-stage method with CUSUM and random forest algorithms. Water Supply.

[22] Li G., Wang J., Liang J., Yue C. (2018). The application of a double CUSUM algorithm in industrial data stream anomaly detection. Symmetry.

[23] Meng M., Wang L., Shang W. (2018). Decomposition and forecasting analysis of China’s household electricity consumption using three-dimensional decomposition and hybrid trend extrapolation models. Energy.

[24] Colonna M., Danzon A., Delafosse P., Mitton N., Bara S., Bouvier A.-M., Ganry O., Guizard A.-V., Launoy G., Molinie F. (2008). Cancer prevalence in France: Time trend, situation in 2002 and extrapolation to 2012. Eur. J. Cancer.

[25] Ma J., Ding Y., Cheng J.C.P., Jiang F., Wan Z. (2019). A temporal-spatial interpolation and extrapolation method based on geographic Long Short-Term Memory neural network for PM_2.5_. J. Clean. Prod..

[26] Liang H., Song L., Wang J., Guo L., Li X., Liang J. (2021). Robust unsupervised anomaly detection via multi-time scale DCGANs with forgetting mechanism for industrial multivariate time series. Neurocomputing.

